# Resistance monitoring and mechanism in the fall armyworm *Spodoptera frugiperda* (Lepidoptera: Noctuidae) for chlorantraniliprole from Sichuan Province, China

**DOI:** 10.3389/fphys.2023.1180655

**Published:** 2023-05-05

**Authors:** Hui-Lin Chen, Ali Hasnain, Qing-Hua Cheng, Li-Juan Xia, Yu-Hao Cai, Rong Hu, Chang-Wei Gong, Xue-Mei Liu, Jian Pu, Lei Zhang, Xue-Gui Wang

**Affiliations:** ^1^ State Key Laboratory of Crop Gene Exploration and Utilization in Southwest China, Sichuan Agricultural University, Chengdu, China; ^2^ College of Agriculture, Sichuan Agricultural University, Chengdu, China; ^3^ College of Plant Protection, Nanjing Agricultural University, Nanjing, China; ^4^ Key Laboratory of Integrated Pest Management on Crops in Southwest, Sichuan Academy of Agricultural Sciences, Ministry of Agriculture, Institute of Plant Protection, Chengdu, China; ^5^ Talent Development Service Center, Sichuan Provincial Department of Agriculture and Rural Affairs, Chengdu, China; ^6^ Department of Entomology, China Agricultural University, Beijing, China

**Keywords:** Spodoptera frugiperda, chlorantraniliprole, cytochrome P450, qRT-PCR, resistance mechanism

## Abstract

The fall armyworm, *Spodoptera frugiperda* (Noctuidae: Lepidoptera), is a wide-reaching notorious insect pest of important cereal crops, which has developed resistance to multiple classes of insecticides. It invaded the Sichuan Province of China in 2019. In this study, we performed resistance monitoring of insecticides for 11 field-collected populations from Sichuan, and all the populations were susceptible to emamectin benzoate and chlorpyrifos. The variations in resistance level to indoxacarb (resistance ratio (RR), 9.23–45.53-fold), spinetoram (RR, 4.32–18.05-fold), and chlorantraniliprole (RR, 2.02–10.39-fold) were observed among these populations. To investigate the resistance mechanism of chlorantraniliprole, synergism tests were performed and showed that piperonyl butoxide had a slight synergistic effect on chlorantraniliprole for the QJ-20 population (1.43-fold) in moderate resistance (RR, 10.39-fold) compared with the treatment group without synergist. Furthermore, the expression scanning for resistance-related genes showed that five P450 genes (*CYP6AE43, CYP321A8, CYP305A1, CYP49A1*, and *CYP306A1*) and the ryanodine receptor gene (*Ryr*, chlorantraniliprole target) were overexpressed in the QJ-20 population. These results indicated that the fall armyworm in Sichuan has exhibited diverse susceptibilities to several classes of insecticides, and the overexpression of *Ryr* and several P450 genes may contribute to the development of resistance in *S. frugiperda* to chlorantraniliprole.

## 1 Introduction

The fall armyworm (FAW), *Spodoptera frugiperda* (Noctuidae: Lepidoptera), is an instinctive pest of the tropical regions of the continents of North and South America, which has rapidly spread over a large area (Sparks et al., 1979). Being polyphagous, it is a notorious pest to more than 350 plant species, including maize, cotton, sugarcane, wheat, sorghum, rice, tomato, beet, and pasture grasses (Montezano et al., 2018). There are two host plant strains of this insect pest, the “corn-biotype” (C-biotype) mostly feeds on maize, sorghum, and cotton, while the “rice-biotype” (R-biotype) mostly targets rice and many other pasture grasses (Dumas et al., 2015).

Among the control methods for agricultural insect pests, chemical control is widely preferable in the farming community across the world. Common pesticides include organophosphorus, carbamate, pyrethroid, and benzoylurea insecticides, which have been supplemented by newer insecticides (indoxacarb, chlorantraniliprole, and emamectin benzoate) in recent years. Owing to the overuse of insecticide, the resistance ratios (RRs) of several FAW populations collected from Mexico and Puerto Rico to permethrin, chlorpyrifos, chlorantraniliprole, and flubendiamide were up to 500-fold in 2016 and were similar for methomyl, deltamethrin, and cypermethrin (Gutierrez et al., 2019). It was reported that the FAW populations invading China carried resistance to the pyrethroids and organophosphates insecticides (Zhang B. et al., 2020). However, most FAW populations in China were found to be susceptible to emamectin benzoate, chlorantraniliprole, spinetoram, indoxacarb, lambda-cyhalothrin, and acephate (Wang et al., 2022). The FAW had no previous record of being resistant to chlorantraniliprole during 2019–2021 (Liu et al., 2022; Wang et al., 2022). However, compared with the 2019 field-collected population, several FAW field populations (collected during 2020) showed slightly reduced toxicity in chlorantraniliprole (Wang et al., 2022), which is a broad spectrum, has high insecticidal efficacy, and is capable of controlling lepidopteran pests. Meanwhile, using less-harmful insecticides inappropriately for a brief period of time in the field would enhance the selection pressure on the target pests and raise the probability of resistance development (Li et al., 2021). The key method by which the FAW develops insecticide resistance is the enhanced detoxifying metabolism of pesticides, which is primarily caused by inadequate pesticide treatment. Previous studies on the FAW revealed that the overexpression of glutathione *S*-transferases (GSTs), cytochrome P450s (P450s), and esterases (ESTs) was mainly involved in developing resistance against pyrethroids, organophosphorus, and carbamate pesticides (Liu et al., 2022). In this regard, the application of different synergists can also be helpful to compare their activities under different conditions as these are crucial in the development of resistance against insecticides (Mohan and Gujar, 2003). There are numerous studies on general P450 functions in pesticide resistance. According to reports, insecticide adaptability is associated with known detoxification families, like P450 monooxygenases (Feyereisen et al., 2012). Comparative genomics analysis displayed that the cytochrome P450 gene family has vastly expanded to 425 members in the FAW, of which 283 genes are specific to the FAW (Gui et al., 2022). A comparison of the expression levels of several cytochrome P450 genes using quantitative real-time PCR (qRT-PCR) in the field-collected populations could also be helpful to understand the nature of the resistance mechanism (Lao et al., 2015). According to certain studies, the overexpression of some P450 genes may be the primary cause of the increase in P450 activity, which can contribute to the FAW’s resistance to chlorantraniliprole (Gong et al., 2013; Elzaki et al., 2016). The transcriptomic and genomic studies of the FAW revealed that approximately 117–425 P450 genes in the FAW, including *CYP321A8*, *CYP321A9*, and *CYP321B1*, may play a critical role in insecticide detoxification and, thus, might be involved in pesticide resistance (Zhang D. D. et al., 2020; Chen and Palli, 2021).

There is currently limited literature on resistance monitoring in the FAW from China. It is urgently necessary to gain scientific and practical knowledge about resistance monitoring and to comprehend the underlying mechanisms of insecticide resistance in the FAW to design Integrated Pest Management (IPM) or Insect Resistance Management (IRM). In this study, we examined the resistance levels of 11 *S. frugiperda* field populations from Sichuan Province against emamectin benzoate, chlorpyrifos, spinetoram, chlorantraniliprole, and indoxacarb. In addition, we determined the synergistic effects of three different synergists and compared the detoxification enzymes’ activities under different treatments. Moreover, we observed the relative expression levels for 26 resistance-related genes (one ryanodine receptor gene (*Ryr)* and 25 P450 genes) in the field population by qRT-PCR. Therefore, the results of our study may be useful in planning appropriate management strategies for the resistant field populations of the FAW, mainly against chlorantraniliprole, and provide a theoretical basis for exploring the extent of resistance and its mechanism against different insecticides used for controlling *S. frugiperda*.

## 2 Materials and methods

### 2.1 Insects

**FIGURE 1:**
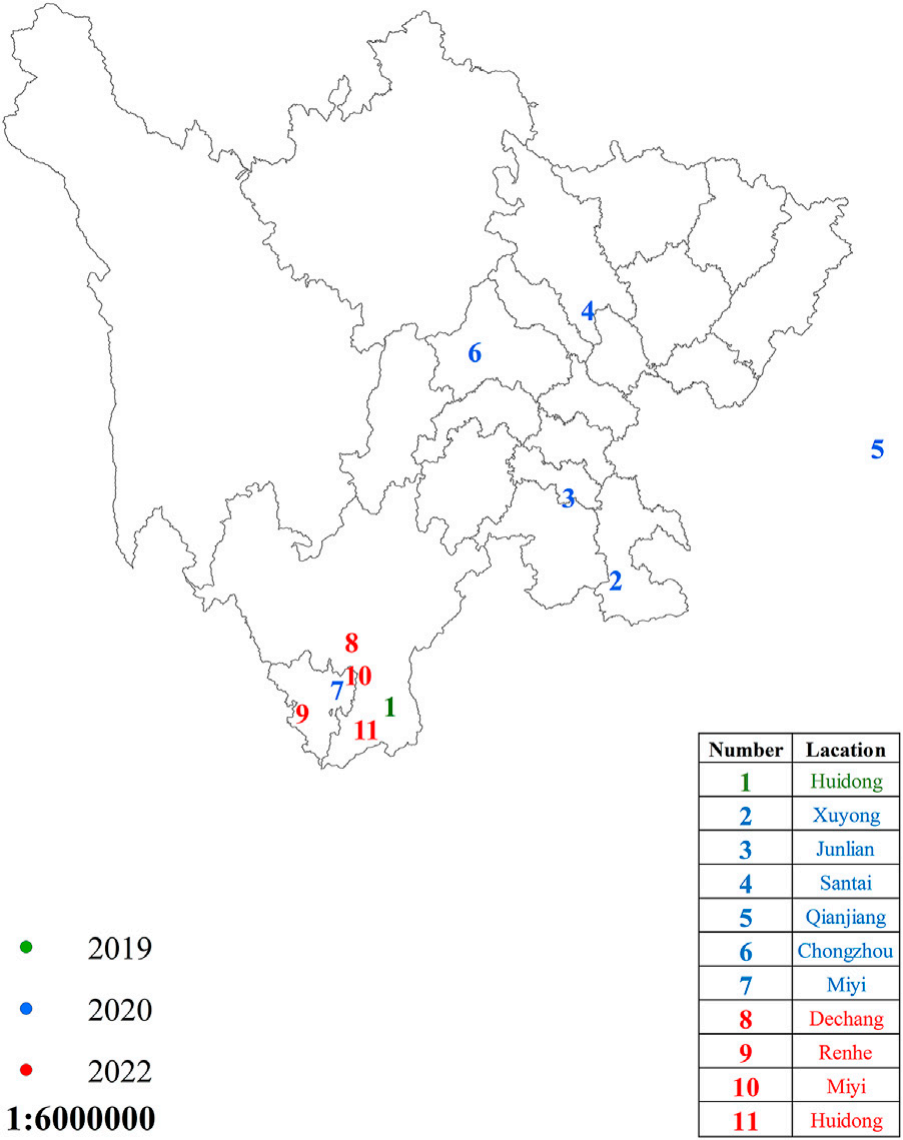
*Spodoptera frugiperda* sampling regions in Sichuan Province, China. The dots in green, blue, and red represent areas where *S. frugiperda* were sampled during the years 2019, 2020, and 2022, respectively.

The 11 populations of the FAW were collected from different places in Sichuan Province, China, in 2019–2022 (Figure 1). The larvae were reared on artificially prepared food. The newly hatched larvae were shifted into small boxes containing food, and when they reached the third instar, they were shifted individually into small glass tubes with artificial food to avoid cannibalism, which is highly reported in the FAW (Chapman et al., 2001). The adults were reared on a 10% honey water solution. The newly laid eggs and pupae were treated with sodium hypochlorite disinfection solution (0.2–0.3%) to avoid any contamination (Wang et al., 2018). All the developmental stages were kept under the controlled conditions of 70–80% relative humidity (RH), 26 ± 1 °C temperature, and a 16:8 h (L:D) photoperiod.

### 2.2 Insecticides and chemicals

Five different insecticides were used in this research, including 74% emamectin benzoate (Nanjing Red Sun Co., Ltd., Gaochun, China), 97% chlorpyrifos (Hubei Sharonda Co., Ltd., Jingzhou, China), 95% chlorantraniliprole (Corteva Agriscience, Indianapolis, United States), 95% indoxacarb (Jiangsu Flag Chemical Industry Co., Ltd., Nanjing, China), and 99% spinetoram (Wuhan Xiyu Biotechnology Co., Ltd., Hubei, China). The synergists included diethyl maleate (DEM), piperonyl butoxide (PBO), and triphenyl phosphate (TPP), which were bought from Aladdin Shanghai Co., Ltd. (Nanjing, China). α-Naphthyl acetate (α-NA), 1-chloro-2,4-dinitrobenzene (CDNB), and fast blue salt B were purchased from Chengdu Ai Keda Chemical Technology Co., Ltd. (Chengdu, China). Bovine serum albumin (BSA), NADPH, DTT, PMSF, and reduced glutathione (GSH) were purchased from Beijing Solarbio Science and Technology Co., Ltd. (Beijing, China). The Coomassie brilliant blue G 250, EDTA Na_2_, and sodium dodecyl sulfate (SDS) were bought from Chengdu Kelong Chemical Reagent Co., Ltd. (Chengdu, China).

### 2.3 Bioassays

For the bioassays, the topical application method was used for 2 days after the third instar larva molting (Wang et al., 2021). There were three replications for each concentration with 12 larvae per replication. The stock solutions for all insecticides were prepared using acetone or dimethyl sulfoxide and then serially diluted to make five or six concentrations of each, to cover mortality from 0 to 100%. A 50 μL microsyringe (Hamilton company, Reno, NV) coupled with a microapplicator (PB600-1 Repeating Dispenser, Hamilton Company) was used to apply 1 μL of each prepared concentration over the dorsal side of the frontal thorax of the third instar larva, as reported in previous reports (Brewer and Trumble, 1989; Mccaffery et al., 1991). We selected the 23rd instar larvae in the same condition to weigh and finally figure out 0.006 g/larva. One microliter of acetone per larva was applied or dimethyl sulfoxide was used for the control treatment. The treated larvae were transferred in 12-compartment culture plates, containing a small quantity of food. Each culture plate was considered one replication. The mortality was assessed 24 h after treatment. If they showed severe intoxication symptoms, including feeding cessation, severe growth inhibition or slow movement, and twitching when touched with a small brush, the larvae were considered dead.

The toxicity of synergists including DEM, TPP, and PBO with chlorantraniliprole was determined for the QJ-20 population using topical application, as previously mentioned. All these synergists were dissolved in acetone at a concentration of 1000 mg/L (Sun et al., 2018). After this, a 1.0 µL droplet was applied over the thoracic dorsum of each third instar larva using a microsyringe coupled with a microapplicator (PB600-1 Repeating Dispenser, Hamilton Company). After an hour of synergist application, the different concentrations of insecticide were applied over larvae, as described previously, and mortality was observed 24 h after treatment.

### 2.4 Enzymatic assays

The activities of CarEs, GSTs, and P450s were determined for the three experimental groups including the QJ-20 population, the QJ-20 population treated only with a synergist, and the QJ-20 population treated with LD_25_ of chlorantraniliprole plus a synergist (TPP or DEM or PBO) (Liu et al., 2022). The fifth instar larvae were selected, and their midguts were dissected over dry ice packs (Wang et al., 2018). These midguts were first put in 1.15% ice-cold KCl solution to isolate the excessive fatty material and were then transferred to a 1.5 mL centrifuge tube, labeled, and stored at −80 °C as quickly as possible.

The determination of CarEs activity was made according to Van Asperen (1962). First, 1 mL of buffer solution (0.04 mol/L phosphate buffer, pH 7.0) was added in a 5 mL glass homogenizer to homogenize the midguts on ice, and then, centrifugation (10000 ×*g* for 10 min at 4 °C) was performed, using a 5417R centrifuge (Eppendorf, Germany). Later, the supernatant was transferred to a clean tube, considered an enzyme source, and then, 1.8 mL of α-NA solution (having 3 × 10^−4^ mol/L physostigmine), 0.45 mL of buffer solution (0.04 mol/L, pH 7.0), and 50 μL of enzyme source were put in the 4 mL centrifuge tube and incubated in a water bath for 15 min at 30 °C. Next, 0.9 mL of staining solution (with 0.2 g of fast blue salt B in 20 mL distilled water plus 50 mL of 5% SDS) was added to stop the reaction. The changes in absorbance values were recorded (after 5 min of calibration with blank) at a wavelength of 600 nm, using the UV 2000 spectrophotometer (Unic Instruments Incorporated, Shanghai, China). The CarEs activity was expressed as millimoles of naphthol per minute per milligram of protein and calculated from the production of α-naphthol by referring to an experimentally determined standard curve.

The GST activity was determined according to Habig and Jakoby (1981), for which the midguts of the fifth instar larvae were first homogenized over ice using 1 mL of buffer (0.1 mol/L phosphate buffer, having 1 mmol/L EDTA, pH 6.5), and the supernatant was transferred to the new tube after centrifugation at 10800 ×g for 10 min at 4 °C, the supernatant was considered as an enzyme source. Next, 90 µL of CDNB (15 mmol/L), 90 µL of reduced GSH (30 mmol/L), 2470 µL of phosphate buffer (0.1 mol/L, pH 6.5), and 50 µL of enzyme source were mixed in a 4 mL tube. The OD value was recorded for 2 min at 340 nm and calculated as △A^340^/min.

The method of Rose et al. (1995) was used to determine the activity of cytochrome P450s ethoxycoumarin *O*-deethylase (ECOD), with some modifications. The enzyme source was the supernatant of the homogenized midguts of the fifth instar larvae, obtained by adding 1 mL of buffer (0.1 mol/L phosphate buffer, pH 7.5, having 20% glycerol, 0.1 mmol/L DTT, 0.1 mmol/L EDTA, and 0.4 mmol/L PMSF), in a 5 mL glass homogenizer over the ice, and centrifugation was conducted at 10000 ×*g* for 15 min at 4 °C. Later, 90 μL of enzyme source was mixed with 100 μL of 2 mmol/L 4-nitroanisole and put into the culture plate and incubated at 27 °C for 3 min in the water bath. After this, 10 μL of 9.6 mmol/L NADPH was added to initiate the reaction, and the absorbance values at 405 nm for 2 min with 20 s intervals at 27 °C were determined using the microplate reader (Model 680 Microplate Reader, Bio-Rad).

The method of Bradford (1976) was used to determine the total protein content, considering BSA as a standard. A test solution of 3 mL was prepared by adding a certain prepared concentration of BSA with 2.5 mL of Coomassie Brilliant blue to measure the OD value at a wavelength of 595 nm using the UV 2000 Spectrophotometer (Unic Instruments Incorporated, Shanghai, China) to get the standard curve.

### 2.5 qRT-PCR

A total of 26 genes were chosen from Giraudo et al. (2014) and the NCBI database to determine which genes contribute to the development of the FAW’s resistance to chlorantraniliprole, and we designed their corresponding primers using Primer-BLAST on the NCBI website (Supplementary Table S1). The midgut of the fifth instar larvae was obtained by dissection on dry ice packs (Wang et al., 2018). RNA was examined, and cDNA synthesis and sequencing of genes were carried out. Due to the lack of a susceptible strain, the relative expressions of all the genes were later discovered in the QJ-20 population, a moderately resistant population (among the 11 populations), using the comparatively sensitive population (HD-19) of *S. frugiperda* as the control. Six higher-expression genes were screened out and were further determined in the six field populations collected in 2020 using qRT-PCR.

The fifth instar larvae were selected for the extraction of RNA using RNase Easy Water (Vazyme Biotech Ltd., China) according to the manufacturer’s instructions, and later, the concentrations of RNA were measured using the Micro-Drop ultra microspectrophotometer (Baoyide Scientific Ltd., Shanghai, China). The Novoscript^®^ plus all-in-one reagent kit (Novoprotein Scientific Inc., Shanghai, China) was used to prepare cDNA from 1 μg total RNA. The Applied Biosystems 7500 RT-PCR system (Applied Biosystems, Foster City, CA, United States) was used to perform qRT-PCR using the 2 × NovoStart^®^ SYBRqPCR SuperMix Plus kit (Novoprotein Scientific Inc.). The total volume of the reaction mixture was 20 μL with 10 μL of 2 ×SuperMix, 1.5 μL of each primer, 1 μL of cDNA template, and 6 μL of RNase-free water. The protocol program was as follows: 95 °C for 1 min, followed by 40 cycles of 95°C for 20 s, 60°C for 20 s, and 72°C for 30 s. The reference gene was the elongation factor 1 alpha (*EF1α*) for *S. frugiperda* (Shu et al., 2020), and the experiment was performed for three technical replicates with three independent biological replicates. The 2^−ΔΔCt^ method (Livak and Schmittgen, 2001) was used for calculating the relative expression levels of target genes.

### 2.6 Data analysis

The POLO 2.0 program (Leora Software, www.leorasoftware.com) was used to calculate the slope, LD_50_, 95% confidence intervals (CI), and chi-square (χ^2^) value of each insecticide 24 h after treatment (Wang et al., 2018). RRs were calculated using the susceptible baseline values for indoxacarb, emamectin benzoate, chlorantraniliprole, and spinetoram as the factor divisor from the result by Wang et al. (2022), while for chlorpyrifos, the most susceptible population was selected as the reference baseline. The resistance levels for insecticides were classified as susceptible (RR *˂* 5.0), low level of resistance (5.0 ≤ RR *˂* 10.0), moderate level of resistance (10.0 ≤ RR *˂* 100.0), and high level of resistance (RR ≥ 100.0) (Lu et al., 2017; Zhang et al., 2017). The activities of CarEs, GSTs, and P450s along with the relative normalized expression of resistance-related genes were expressed as the mean ± standard error (SE), compared using the analysis of variance (ANOVA) followed by Tukey’s test (*p ˂* 0.05) with the SPSS version 17.0 software package (IBM), while the graphs were prepared using the mean with SE in the Sigma Plot 10.0 software. Correlation analysis between the relative expressions of *Ryr*, P450 genes, and chlorantraniliprole resistance in the field populations of *S. frugiperda* in 2020 was calculated according to the Pearson method using the SPSS Statistics software package (Lu et al., 2017). A *p*-value of *p* < 0.05 or *p* < 0.01 was considered to be statistically significant or extremely significant.

## 3 Results

### 3.1 Susceptibility status and variations in field populations

The toxicities of five insecticides toward the field populations are given in Table 1. Field populations displayed low levels of resistance to chlorpyrifos. For chlorpyrifos, their LD_50_ values ranged from 106.680 μg/g (RH-22) to 339.518 μg/g (MIYI-22). For chlorantraniliprole, there was a deviation among the field populations whose RR values ranged from 2.02-fold (HD-19) to 10.39-fold (QJ-20), and their resistance levels ranged from susceptible to moderate level, and the QJ-20 population had the highest LD_50_ value (4.260 μg/g). For spinetoram, their RR values were ranging from 4.32-fold (DC-22) to 18.05-fold (RH-22). The LD_50_ values of all the different populations to emamectin benzoate were distributed between 0.242 μg/g (HD-19) and 0.886 μg/g (MIYI-22), and their RR values were ranging from 0.68-fold to 2.44-fold. According to insecticides of the sensitive baseline, the resistance of spinetoram was monitored in 2022, and only four populations were calculated. The LD_50_ values ranged from 2.197 μg/g to 10.837 μg/g with an RR of 9.23-fold (HD-22) to 45.53-fold (DC-22).

**TABLE 1:** Resistance levels of the field-collected populations of fall armyworm *Spodoptera frugiperda* to several insecticides in the topical bioassay method.

Insecticide	Population	Slope ±SE	LD_50_ (µg.g ^−1^)	95% CI (µg/g)^a^	χ^2^ (df)^b^	p^*^	RR
Chlorantraniliprole	SUS **	1.139 ± 0.234	0.410	0.229–0.602	15.42 (18)	0.6320	1.00
	HD-19	1.335 ± 0.329	0.830	0.137–1.708	4.52 (10)	0.9208	2.02
	XY-20	1.503 ± 0.269	1.651	0.695–2.711	6.35 (13)	0.9324	4.03
	JL-20	1.506 ± 0.285	1.408	0.535–2.383	7.66 (13)	0.8650	3.43
	ST-20	1.813 ± 0.337	1.807	0.901–2.732	6.94 (10)	0.7310	4.41
	QJ-20	1.768 ± 0.275	4.260	2.755–5.906	7.02 (10)	0.7236	10.39
	CZ-20	1.609 ± 0.288	1.607	0.708–2.586	6.23 (13)	0.9374	3.92
	MIYI-20	1.780 ± 0.309	1.770	0.886–2.702	5.52 (13)	0.9619	4.32
	DC-22	0.813 ± 0.157	1.838	0.623–3.376	11.31 (16)	0.7313	4.48
	HD-22	0.837 ± 0.157	1.910	0.689–3.438	8.22 (16)	0.8376	4.66
	MIYI-22	0.818 ± 0.154	2.812	1.161–4.853	10.79 (16)	0.9658	6.86
	RH-22	1.518 ± 0.201	2.536	1.546–3.662	16.12 (16)	0.8243	6.19
Emamectin benzoate	SUS **	1.830 ± 0.273	0.355	0.275–0.465	12.20 (18)	0.8370	1.00
	HD-19	2.145 ± 0.348	0.242	0.148–0.325	7.11 (16)	0.9711	0.68
	XY-20	3.810 ± 0.507	0.760	0.635–0.876	9.27 (13)	0.7522	2.14
	JL-20	3.038 ± 0.472	0.646	0.496–0.774	9.20 (13)	0.7576	1.82
	ST-20	4.073 ± 0.547	0.688	0.572–0.793	6.59 (13)	0.9225	1.94
	QJ-20	3.528 ± 0.534	0.574	0.446–0.683	8.49 (13)	0.8103	1.62
	CZ-20	3.457 ± 0.481	0.781	0.616–0.932	15.39 (13)	0.2836	2.20
	MIYI-20	2.919 ± 0.474	0.603	0.448–0.732	12.19 (13)	0.5121	1.70
	DC-22	1.016 ± 0.180	0.327	0.162–0.528	8.83 (13)	0.9447	0.92
	HD-22	1.397 ± 0.117	0.543	0.36–0.777	6.29 (13)	0.9104	1.53
	MIYI-22	1.368 ± 0.183	0.886	0.614–1.275	8.47 (13)	0.9557	2.44
	RH-22	1.036 ± 0.168	0.659	0.392–1.028	7.44 (13)	0.9432	1.86
Chlorpyrifos	RH-22(SUS *)	4.604 ± 0.544	106.680	94.958–122.008	8.38 (16)	0.9367	1.00
	HD-19	3.429 ± 0.342	113.680	99.719–130.333	7.94 (19)	0.9872	1.07
	XY-20	5.111 ± 0.591	122.309	108.919–136.648	3.71 (16)	0.9993	1.15
	JL-20	4.587 ± 0.524	149.808	131.630–169.454	9.47 (16)	0.8928	1.40
	ST-20	3.027 ± 0.341	135.578	115.128–159.575	7.93 (16)	0.9509	1.27
	QJ-20	4.582 ± 0.590	108.769	95.778–125.099	8.50 (13)	0.8095	1.02
	CZ-20	4.175 ± 0.484	113.701	100.362–131.351	8.94 (16)	0.9158	1.07
	MIYI-20	1.77 ± 0.175	122.074	71.764–233.539	4.27 (16)	0.9141	1.14
	DC-22	0.852 ± 0.233	150.399	88.847–460.269	1.86 (13)	0.9120	1.41
	HD-22	1.722 ± 0.230	179.766	95.600–1109.614	1.35 (13)	0.7125	1.69
	MIYI-22	1.047 ± 0.258	339.518	186.341–1455.745	6.69 (13)	0.7053	3.18
Indoxacarb	SUS **	2.147 ± 0.300	0.238	0.186–0.304	11.22 (18)	0.9170	1.00
	HD-19	2.090 ± 0.300	4.292	2.967–5.748	6.64 (10)	0.7589	18.03
	XY-20	2.155 ± 0.325	3.221	2.159–4.364	8.51 (10)	0.5791	13.53
	JL-20	1.889 ± 0.250	5.333	3.684–7.144	10.81 (13)	0.6267	22.41
	ST-20	2.000 ± 0.291	5.162	3.606–6.948	5.20 (10)	0.8774	21.69
	QJ-20	2.005 ± 0.288	5.492	3.873–7.316	8.68 (10)	0.5627	23.08
	CZ-20	1.608 ± 0.266	3.921	2.365–5.604	5.90 (10)	0.8235	16.47
	DC-22	1.965 ± 0.239	10.837	8.388–13.813	9.92 (16)	0.9535	45.53
	HD-22	1.174 ± 0.177	2.197	1.177–3.372	14.31 (16)	0.8529	9.23
	MIYI-22	1.111 ± 0.165	3.521	2.036–5.280	14.40 (16)	0.8091	14.79
	RH-22	1.506 ± 0.216	3.050	1.990–4.256	10.12 (13)	0.8220	12.82
Spinetoram	SUS **	2.162 ± 0.261	0.518	0.416–0.651	12.20 (22)	0.7950	1.00
	DC-22	1.112 ± 0.172	2.240	1.061–3.634	16.48 (16)	0.9006	4.32
	HD-22	1.181 ± 0.166	9.095	5.782–14.005	18.69 (16)	0.8955	17.56
	MIYI-22	1.483 ± 0.185	5.870	4.129–7.957	11.75 (16)	0.9088	11.33
	RH-22	1.324 ± 0.175	9.349	6.617–13.010	14.91 (16)	0.9502	18.05

The median lethal dose (LD_50_) is expressed as micrograms of active ingredient per gram of insect.

SUS*: The most susceptible population among the field populations was considered the susceptible baseline.

SUS**: The susceptibility baseline data were referred from the work of Wang et al., 2022.

^a^ RR, resistance ratio, calculated as LD_50_ of field population/LD_50_ susceptibility baseline.

^b^Chi-square value (*χ* ^2^) and degrees of freedom (df) as calculated using Probit analysis (Polo Plus 2.0).

### 3.2 Synergism of TPP, PBO, and DEM

The effects of TPP, PBO, and DEM on the toxicity of chlorantraniliprole toward the QJ-20 population (RR = 10.39-fold) are given in Table 2. PBO had the highest synergistic of chlorantraniliprole for the QJ-20 population in moderate resistance (RR = 10.39-fold) compared with the treatment group without a synergist. The synergists PBO and DEM showed a slight increase in the toxicity of chlorantraniliprole, among which PBO enhanced the efficacy up to 1.43-fold and its LD_50_ treated by PBO decreased from 0.716 μg/g to 0.500 μg/g, while for DEM (LD_50_ = 0.667 μg/g) and TPP (LD_50_ = 0.767 μg/g), the synergistic ratios were only 1.08- and 0.93-fold, respectively.

**TABLE 2:** Synergism of DEM, TPP, and PBO with chlorantraniliprole in the QJ-20 population of *S. frugiperda.*

Treatment	Slope ±SE	LD_50_ (µg.g ^−1^)^a^ (95% CI)	χ^2^ (df)	p^*^	SR^b^
Chlorantraniliprole	2.240 ± 0.320	0.716 (0.533–0.933)	15.09 (10)	0.7236	1
+ DEM	2.678 ± 0.349	0.667 (0.533–0.800)	10.56 (13)	0.6467	1.08
+ TPP	2.720 ± 0.356	0.767 (0.633–0.917)	12.87 (13)	0.4579	0.93
+ PBO	2.529 ± 0.363	0.500 (0.367–0.617)	12.08 (13)	0.5210	1.43

^a^ The median lethal dose (LD_50_) expressed as micrograms of active ingredient per gram of insect.

^b^ SR (synergist ratio) = LD_50_ of synergist plus insecticide/LD_50_ of the insecticide.

^*^Goodness-of-fit test is significant at *p* > 0.05.

### 3.3 Detoxification enzyme activities

To evaluate the role of these three detoxification enzymes in the development of resistance in *S. frugiperda* against chlorantraniliprole, the activities of CarEs, GSTs, and P450s were determined. As shown in Table 3, there was no significant difference among the activities of CarEs for all treatments (from 0.282 to 0.519 mmol/min. mg pro) (*p* ˃ 0.05). Similarly, no significant difference was observed among the activities of GSTs with the values of 0.106–0.166 mmol/min. mg pro, respectively (*p* ˃ 0.05). Meanwhile, for cytochrome P450s, there was significant difference in the activities between the QJ-20 population (0.629 nmol/min. mg pro) and PBO synergist application treatment (0.273 nmol/min. mg pro) (*p* < 0.05), but no significant difference with the treatment of PBO plus LD_25_ of chlorantraniliprole (0.439 nmol/min. mg pro) (*p* ˃ 0.05).

**TABLE 3:** Detoxification enzyme activities of the QJ-20 strain.

Treatment	CarE activity, mmol/min.mg pro	IR^a^	GST activity, mmol/min.mg pro	IR^a^	Cytochrome P450 ECOD activity, nmol/min.mg pro	IR^a^
QJ-20	0.519 ± 0.13 a		0.166 ± 0.01 a		0.629 ± 0.09 a	
QJ-20 treated with a synergist	0.282 ± 0.04 a	0.46	0.106 ± 0.02 a	0.36	0.273 ± 0.03 b	0.57
QJ-20 treated with a synergist plus LD_25_ dosage of chlorantraniliprole	0.426 ± 0.06 a	0.18	0.157 ± 0.02 a	0.05	0.439 ± 0.04 ab	0.30
	F3, 8 = 1.984, *p* < 0.05		F3, 8 = 3.118, p < 0.05		F3, 8 = 8.825, p < 0.05	

The synergists that used CarE, GST, and P450 activities in the populations were TPP, DEM, and PBO, respectively.

^a^ Inhibition ratio (IR) presents the detoxification enzyme activities of QJ-20 treated with a synergist or plus LD_25_ dosage of chlorantraniliprole/the detoxification enzyme activities of QJ-20 treated without a synergist.

^b^ Different letters indicate significant differences (*p* < 0.05) using ANOVA followed by Tukey’s test (*p* < 0.05) with the SPSS version 17.0 software package (IBM).

### 3.4 Relative expressions of *Ryr* and *P450* genes

**FIGURE 2:**
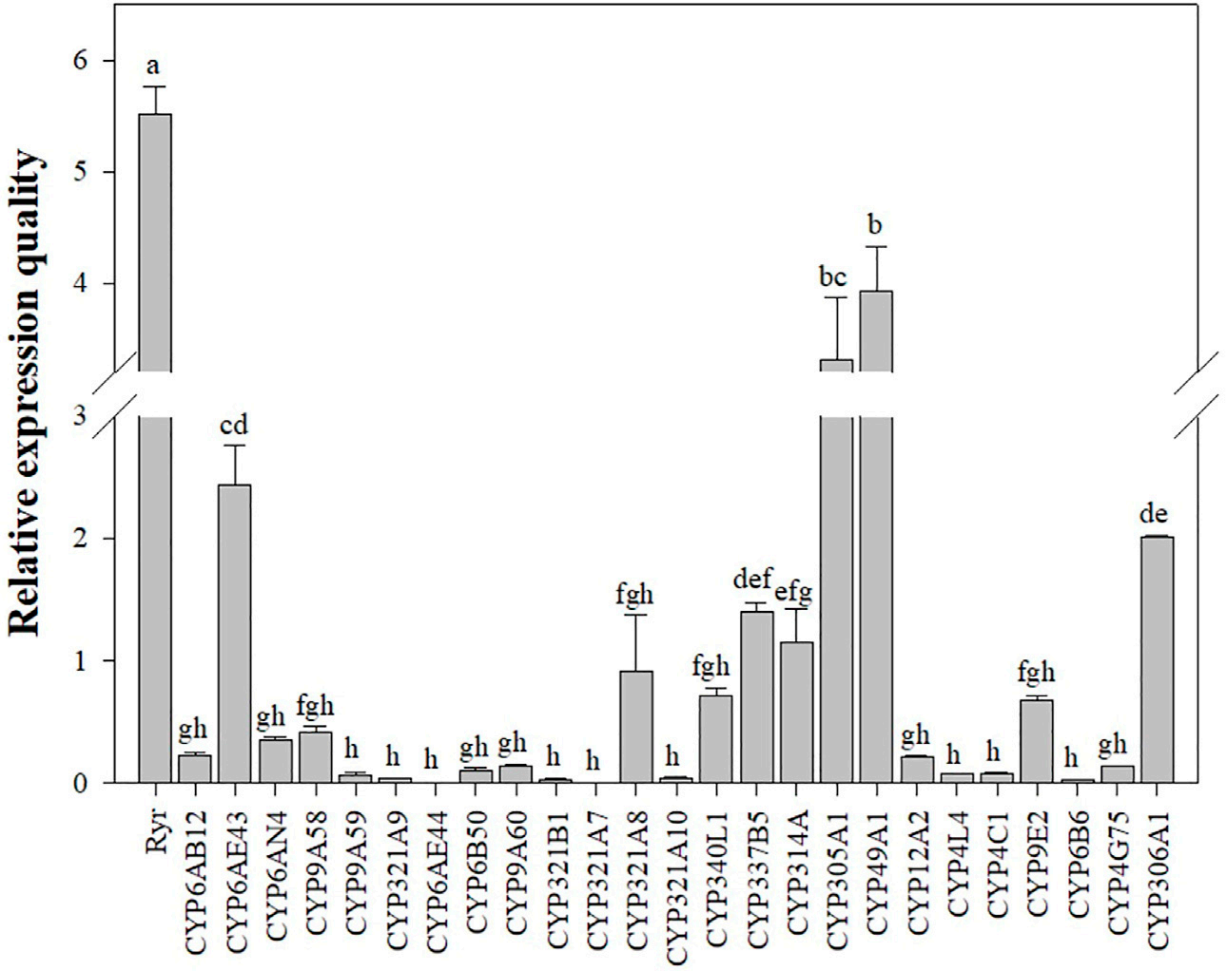
Relative expression levels of the *CYP450* and *Ryr* gene in the QJ-20 population of FAW. The relative expression levels were compared using one-way ANOVA followed by Tukey’s test, at 0.05 level of significance, in SPSS version 17.0 software (IBM). Letters above the bars indicate significant differences (*p* ˂ 0.05), and means followed by the same letters did not differ significantly (*p* ˃ 0.05).

**FIGURE 3:**
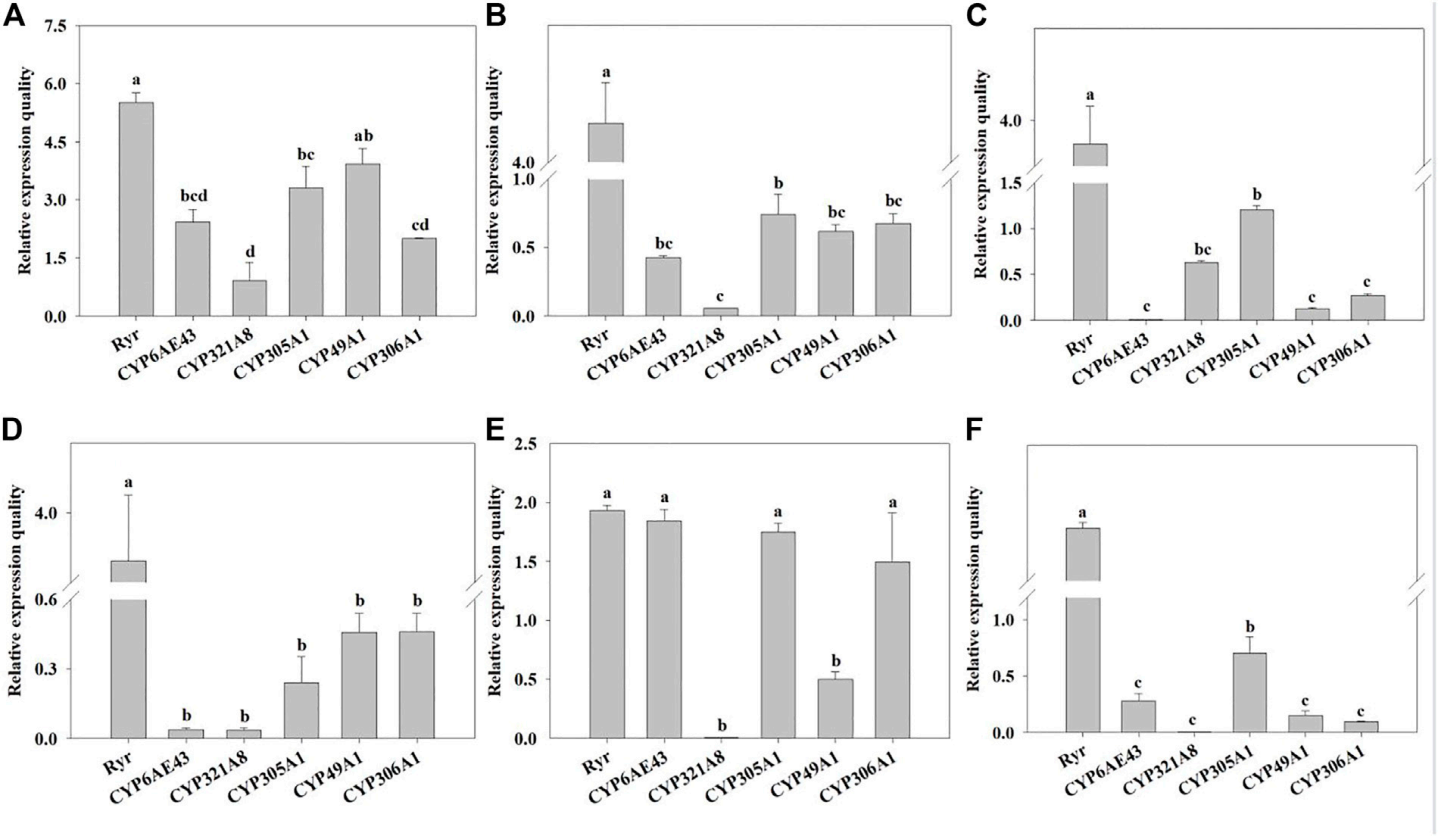
Relative normalized expressions of six resistance-related genes in all field populations. **(A)** QJ-20 field population, **(B)** XY-20 field population, **(C)** JL-20 field population, **(D)** ST-20 field population, **(E)** MIYI-20 field population, and **(F)** CZ-20 field population. Letters above the bars indicate significant differences (*p* ˂ 0.05), and means followed by the same letters did not differ significantly (*p* ˃ 0.05) according to Tukey’s test with SPSS version 17.0 software package (IBM).

The mRNA expression levels of *Ryr* and P450 genes were detected in the QJ-20 population by qRT-PCR (Figure 2). Among these genes, six showed variation with the maximum relative expression for the *Ryr* gene (5.5-fold), followed by *CYP49A1*, *CYP305A1, CYP6AE43, CYP306A1*, and *CYP321A8*, with values of 3.9-, 3.3-, 2.4-, 2-, and 0.9-fold, respectively (Figure 2). Meanwhile, the relative expressions of these five *CYP450* genes and the *Ryr* gene were also calculated in the tested field populations, in which *CYP6AE43*, *CYP321A8, CYP305A1, CYP49A1,* and *CYP306A1* showed relative expression values ranging from 0.009- to 2.4-fold, 0.005- to 0.9-fold, 0.24- to 3.3-fold, 0.12- to 3.9-fold, and 0.9- to 2.0-fold, respectively (Figure 3).

Specifically, the *Ryr* gene showed significantly high relative expression levels, ranging from 1.93- to 5.5-fold, in all field populations (*p* < 0.05), compared with hd-19. Among them, QJ-20 showed the highest expression level (Figure 3). In the populations of XY-20, CZ-20, and JL-20, *Ryr* showed the highest relative expression, followed by *CYP305A1*, with the least significant relative expression of *CYP321A8* in XY-20 (Figure 3B) and CZ-20 (Figure 3F), but in the JL-20 population, the *CYP6AE43* expressed the least significant relative expression (*p* < 0.05) (Figure 3C). For the MIYI-20 population, *Ryr* showed the highest relative expression, followed by *CYP6AE43*, while *CYP321A8* expressed the lowest relative expression (*p* < 0.05) (Figure 3E). Moreover, in the ST-20 population, *Ryr* showed significant overexpression while *CYP321A8* expressed the lowest relative expression (*p* < 0.05) (Figure 3D).

To further verify the relationship between resistance to chlorantraniliprole and the relative gene expressions, we analyzed their correlation for six field populations in 2020. The *Ryr* expression had a correlation with chlorantraniliprole resistance but in a correlation value of 0.810 (*p* > 0.05), and the correlation coefficient of *CYP49A1* and *CYP305A1* reached 0.895 and 0.857 (*p <* 0.05), respectively, while for others, it was <0.8 (*p* > 0.05) (Table 4).

**TABLE 4:** Correlation analysis between the relative expressions of *Ryr*, P450 genes, and chlorantraniliprole resistance in the field populations of *S. frugiperda* in 2020.

	Resistance level	*Ryr*	*CYP6AE43*	*CYP321A8*	*CYP305A1*	*CYP49A1*	*CYP306A1*
Resistance level	1	0.810	0.792	0.245	0.857*	0.895*	0.770
*Ryr*	0.810	1	0.362	−0.143	0.557	0.534	0.401
*CYP6AE43*	0.792	0.362	1	0.658	0.912*	0.972**	0.963**
*CYP321A8*	0.245	−0.143	0.658	1	0.663	0.581	0.668
*CYP305A1*	0.857*	0.557	0.912*	0.663	1	0.930**	0.864*
*CYP49A1*	0.895*	0.534	0.972**	0.581	0.930**	1	0.967**
*CYP306A1*	0.770	0.401	0.963**	0.668	0.864*	0.967**	1

Pearson’s correlation, the correlation coefficient > −1 and <1; the closer it is to −1 or 1, the stronger the correlation between two variables, and the closer it is to 0, the weaker the correlation. *p < 0.05; **p < 0.01.

## 4 Discussion

*Spodoptera frugiperda* is an invasive pest, not only does the unscientific use of chemical insecticides in the field result in the outbreak of resistance but invasive pests themselves may also carry resistance to certain insecticides (Gao et al., 2013). In our study, most tested FAW populations were susceptible to emamectin benzoate, which was consistent with previous reports (Liu et al., 2022; Wang et al., 2022). However, indoxacarb and spinetoram showed medium toxicity and posed a high risk for the evolution of resistance, and the sensitivity to chlorpyrifos had dropped substantially. It might be that those insecticides had still been used in the field, and *S. frugiperda* was also exposed to such agents, resulting in a large LD_50_ value in the field population (Zhang et al., 2021; Zhang et al., 2022). Among them, three field populations of the FAW in Sichuan were in low or moderate resistance to chlorantraniliprole; the resistance level was also increasing with time, and there were potential resistance risks, as reported in previous reports (Wang et al., 2022). This might be due to using unreasonable pesticides, the field selection of the pest, or its migration from other places with high resistance levels.

The resistance mechanism of insects toward insecticides is comprised of two main aspects, including the detoxification enzyme activity upregulation and the target-induced decreased sensitivity (Zhang B. et al., 2020). Low-dose chlorantraniliprole treatment can increase resistance to the same class of insecticides, which suggests that this is the consequence of the induction of particular detoxification enzymes (Riaz et al., 2009). An increase in the detoxification enzymes’ activities, such as CarEs, GSTs, and P450s, is one of the important reasons for the development of resistance toward insecticides (Pedersen et al., 2019), among which enhanced P450s activity plays an essential role (Liu et al., 2006; Mao et al., 2019). Our results also prove that the synergist PBO significantly increased the efficacy of chlorantraniliprole by reducing the P450 enzyme activities in our synergist experiment, and analogous results were also reported by Xu et al. (2019) and Tan et al. (2022), who suggested that P450 might play a primary role in the development of resistance toward chlorantraniliprole. Meanwhile, our result regarding enzyme activity determination also shows that the increase in P450 activity plays an important role in the resistance of *S. frugiperda* to chlorantraniliprole, which is consistent with the previous reports by Zhang D. D. et al. (2020), who reported that the increased activity of P450s enzymes might also be secondarily involved in the resistance mechanism of *S. frugiperda* against chlorantraniliprole.

The overexpression of some P450 genes may be the main reason for the increase in P450 activity, which can referee the resistance or induction of *S. frugiperda t*o chlorantraniliprole (Gong et al., 2013; Elzaki et al., 2016). Our results show that four P450 genes *CYP49A1*, *CYP305A1, CYP6AE43,* and *CYP306A1* were upregulated in the chlorantraniliprole-resistant populations and when exposed to the sublethal concentrations of chlorantraniliprole, *CYP6AE43* in *S. frugiperda* was also upregulated (Xiao and Lu, 2022), while the downregulation of *CYP6AE43* would enhance the susceptibility of *S. frugiperda* to some insecticides (Zhang B. et al., 2020). However, the association analysis between gene expression and chlorantraniliprole resistance revealed that *CYP305A* and *CYP49A1* had the highest correlation with chlorantraniliprole resistance, reaching a significant level. Some studies have reported that pesticides, such as azadirachtin (Li et al., 2015) and phoxim (Yu et al., 2022), could induce the upregulation of *CYP305A* and *CYP49A1* expression. In addition to P450 genes, we also found that the *Ryr* expression was highly correlated with resistance to chlorantraniliprole, which was in support of reports published by Sun et al. (2012) and Qin et al. (2018), who considered that the diamide-resistant populations have higher mRNA expression levels of the *Ryr* gene in *Plutella xylostella*. However, it has also been reported that chlorantraniliprole-resistant populations have lower expression levels of the *Ryr* gene in *P. xylostella* and *Chilo suppressalis* (Gong et al., 2014; Wei et al., 2019). Thus, to investigate whether the aforementioned genes are associated with chlorantraniliprole resistance, in the future, we would design a suitable dsRNA for executing RNA interference (RNAi) or RNA knockout assays to fully elaborate their functions in the development of resistance toward chlorantraniliprole.

Our results show that the resistance of the field populations of *S. frugiperda* in Sichuan toward emamectin benzoate and chlorpyrifos was still at a sensitive level. However, they exhibited a low or moderate level of resistance to chlorantraniliprole. The synergist and detoxification enzyme activity experiment showed that the resistance of *S. frugiperda* against chlorantraniliprole might cause the upregulated activities of the detoxification enzymes. The result of qRT-PCR and the association analysis between gene expression and chlorantraniliprole resistance revealed that the upregulation of *CYP305A1*, *CYP49A1*, and *Ryr* might be related to the upregulated activities of the detoxification enzymes and the induction of particular detoxification enzymes. However, further investigation is imperative to provide functional evidence for a catalytic interaction of chlorantraniliprole, which could include the expression of the corresponding genes *in vivo* or *in vitro* and functional verification by molecular technologies such as RNAi or CRISP-cas9, and so on. Nonetheless, our results provide a foundation for subsequent efforts to control *S. frugiperda* with integrated pest management strategies.

## Data Availability

The original contributions presented in the study are included in the article/Supplementary Material; further inquiries can be directed to the corresponding authors.
