# Supplementary material for: Resistance monitoring and mechanism in the fall armyworm Spodoptera frugiperda (Lepidoptera: Noctuidae) for chlorantraniliprole from Sichuan Province, China
Source: Front Physiol. 2023 May 5;14:1180655. doi: 10.3389/fphys.2023.1180655 (PMC10196208; doi:10.3389/fphys.2023.1180655)
Supplement: Supplementary file 1 [file Table5.pdf]

Table S1 Details of insect-associated genes and their primer sequences.

Gene Accession Number	Primer Name	Primer Sequence	Product Length
**U20139.1**	EF1α F	5’ TGGGCGTCAACAAAATGGA 3’	148
	EF1α R	5’ TCTCCGTGCCAGCCAGAAAT 3’	
**MK226188.1**	Sf RYR F	5’ CACAGGTGGATCTCTCCCAG 3’	142
	Sf RYR R	5’ GCGTCCAACGTAGACACCTT 3’	
**KC789747.1**	Sf CYP6AB12 F	5’ GCTATAGCTGCGGTCCTACTG 3’	167
	Sf CYP6AB12 R	5’ TACAGCTCGTCTGCTACTTGC 3’	
**KJ671575.1**	Sf CYP6AE43 F	5’ GAGCTTACTTCGGCACGTTG 3’	173
	Sf CYP6AE43 R	5’ CAACACTTTCCAGCGGTCG 3’	
**KC789748.1**	Sf CYP6AN4 F	5’ CGCTTGACGCCAACATTTACG 3’	93
	Sf CYP6AN4 R	5’ ATCTTCACCCACTGTCTGCAAT 3’	
**KJ671577.1**	Sf CYP9A58 F	5’ TTCATGAACGAGGCAGTGCT 3’	173
	Sf CYP9A58 R	5’ GGACTTAGTGTGGAGCGCAT 3’	
**KJ671578.1**	Sf CYP9A59 F	5’ GTACGCGACCCAGAACTGATTA 3’	134
	Sf CYP9A59 R	5’ CGCATTTCCTTCCACTCTTTCAT 3’	
**KC789752.1**	Sf CYP321A9 F	5’ AAGTGCTATCAGGTGACTTTCAGT 3’	121
	Sf CYP321A9 R	5’ TTCGATCGCATCAGCTTCCAT 3’	
**KJ671576.1**	Sf CYP6AE44 F	5’ CTTCAGTGGCCGTGAAGTCT 3’	96
	Sf CYP6AE44 R	5’ TGACGTAGGACCTTCCACCT 3’	
**KC789749.1**	Sf CYP6B50 F	5’ ACAATCTTTTCCACGCCGAC 3’	123
	Sf CYP6B50 R	5’ GAAATTGATCCGCTCGGTTGG 3’	
**KJ671579.1**	Sf CYP9A60 F	5’ CTCCAATGGGTTCGAGTCCT 3’	145
	Sf CYP9A60 R	5’ GTCTCCAACTTCCACCATGA 3’	
**KC789754.1**	Sf CYP321B1 F	5’ ACAACGAAAACTACTGGAGGAA 3’	166
	Sf CYP321B1 R	5’ TCGAGTGAGCCCATTCCAAC 3’	
**KC789750.1**	Sf CYP321A7 F	5’ ACACCTGCGCTCTTTGTCAT 3’	113
	Sf CYP321A7 R	5’ TCGCTCAATTGGTCCCCTTC 3’	
**KC789751.1**	Sf CYP321A8 F	5’ GAAGCGTGGCGTAAAGTTCT 3’	162
	Sf CYP321A8 R	5’ AAGAGCGCAGGTGTTAGGAC 3’	
**KC789753.1**	Sf CYP321A10 F	5’ TTGCTACCAATTACTTTGGCCCTCA 3’	163
	Sf CYP321A10 R	5’ GGAACAGGGGACGTGGTGAT 3’	
**KC789755.1**	Sf CYP340L1 F	5’ TGATCGGGCATGTGCCTAAA 3’	165
	Sf CYP340L1 R	5’ TTGCTATCACACTCGCGTCA 3’	

**KJ671580.1**	Sf CYP337B5 F	5’ CAAGCGGTTCTAGCTGGTGA 3’	139
	Sf CYP337B5 R	5’ CTGGACTGAGTTTTTGCCTCA 3’	
**MN480678.1**	Sf CYP314A F	5’ ACGTAGAGAGCTTTTGGGTCG 3’	134
	Sf CYP314A R	5’ AGGTCTGACATCTGCCAACG 3’	
**MN480675.1**	Sf CYP305A1 F	5’ GAGAAAGTATCCGCCGGGTC 3’	100
	Sf CYP305A1 R	5’ ACAAGCTCTCCATTCCGATCC 3’	
**MN480673.1**	Sf CYP49A1 F	5’ AAGAACCCTTGGAAAACCCG 3’	121
	Sf CYP49A1 R	5’ ATGGGAACACTAAGTCGGGG 3’	
**MN480669.1**	Sf CYP12A2 F	5’ TACAGGAGTCAGGAGGCGAA 3’	123
	Sf CYP12A2 R	5’ CTTTTCCCCTTGTGCGGTTG 3’	
**MN480659.1**	Sf CYP4L4 F	5’ TCCTCTGCCCTTAGTGGGAA 3’	159
	Sf CYP4L4 R	5’ TCCTCAGCCTGCTTTGGATG 3’	
**MN480656.1**	Sf CYP4C1 F	5’ ACTTCCTCAAGGGTTGGCTG 3’	134
	Sf CYP4C1 R	5’ CTTCTCCTCGATGACGTGGG 3’	
**MN480668.1**	Sf CYP9E2 F	5’ CAGGAAACCTGTTATATTTGTGGGT 3’	123
	Sf CYP9E2 R	5’ TCAAATATCCCGCCAAAGCG 3’	
**MN480664.1**	Sf CYP6B6 F	5’ TGGGAGTTCATGACGGGAGA 3’	125
	Sf CYP6B6 R	5’ GGATCCTTCACGTAGAGTCCAG 3’	
**MN480658.1**	Sf CYP4G75 F	5’ CTGGACTGCCACCAACTTGT 3’	108
	Sf CYP4G75 R	5’ AGTTTGGCCGCGAGTTCATA 3’	
**XM_035580222.1**	Sf CYP306A1 F	5’ TGTTATCCAAGCAAATTGATCGAG 3’	176
	Sf CYP306A1 R	5’ GTCAAATGCGGCTGATGACG 3’	

The above gene primers were designed by using NCBI Primer BLAST. The elongation factor 1 alpha (*EF1α*) was used as reference gene (Giraudo et al., 2014; Shu et al., 2020).
