# Supplementary figures and images for: Resistance monitoring and mechanism in the fall armyworm Spodoptera frugiperda (Lepidoptera: Noctuidae) for chlorantraniliprole from Sichuan Province, China
Source: Front Physiol. 2023 May 5;14:1180655. doi: 10.3389/fphys.2023.1180655 (PMC10196208; doi:10.3389/fphys.2023.1180655)

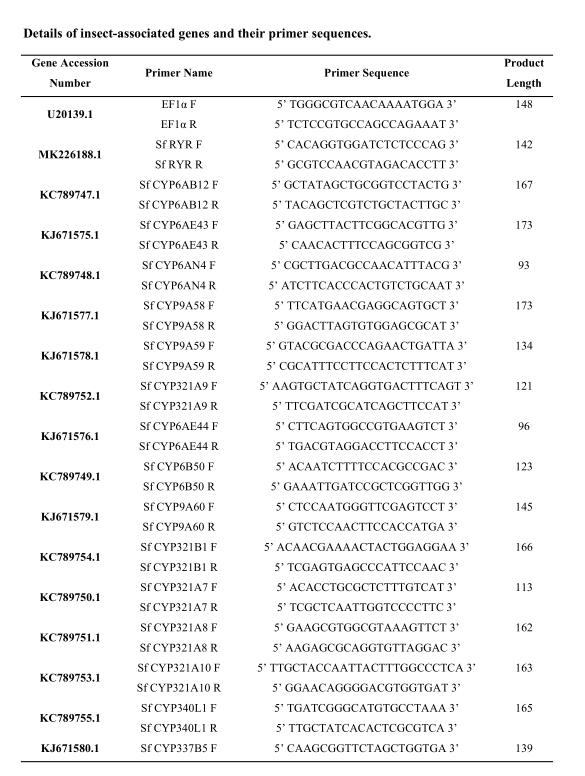

Supplement: Supplementary file 2 [file Image1.JPEG]

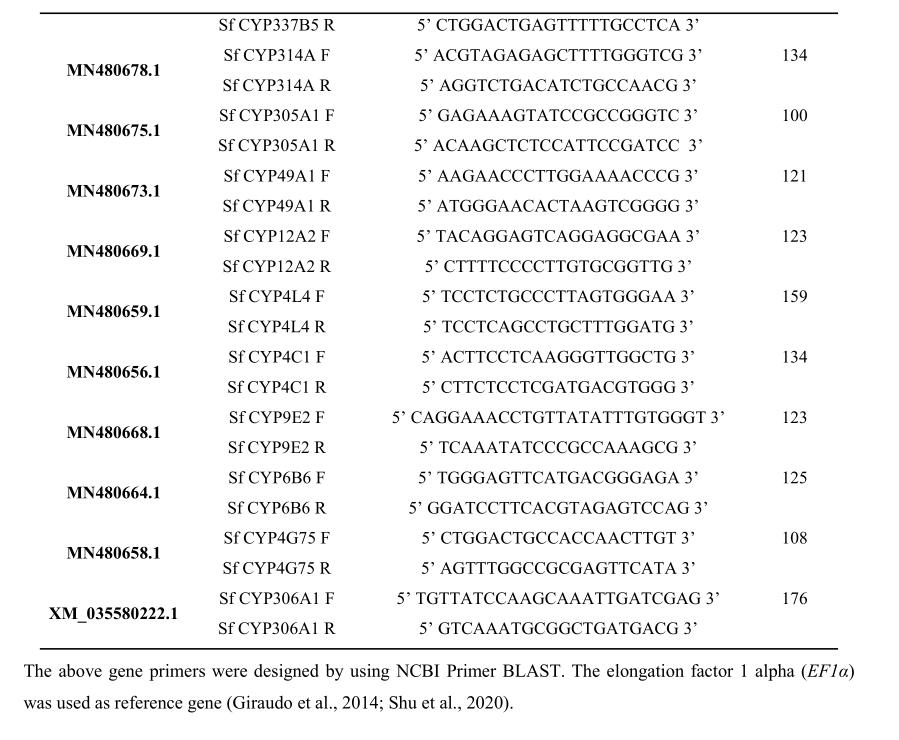

Supplement: Supplementary file 3 [file Image2.JPEG]
